# Belonging in dual roles: exploring professional identity formation among disabled healthcare students and clinicians

**DOI:** 10.1007/s10459-024-10386-4

**Published:** 2024-11-07

**Authors:** Yael Mayer, Laura Nimmon, Michal Shalev, Elisabeth Gross, Laura Yvonne Bulk, Alfiya Battalova, Terry Krupa, Tal Jarus

**Affiliations:** 1 University of Haifa, Haifa, Israel; 2 University of British Columbia, Vancouver, Canada; 3 Ono Academic College, Kiryat Ono, Israel; 4 Royal Roads University, Victoria, Canada; 5 Queen’s University, Kingston, Canada

**Keywords:** Professional identity formation, Belonging, Disability, Healthcare education, Disability identity

## Abstract

The development of a robust professional identity is a pivotal aspect of every healthcare professional’s educational journey. Critical social perspectives are increasingly influencing the examination of professional identity formation within healthcare professions. While understanding how disabled students and practitioners integrate a disability identity into their professional identity is crucial, we have limited knowledge about the actual formation of their professional identity. This study aims to investigate how disabled students and clinicians in healthcare professions actively shape their professional identity during their educational and professional journeys. We conducted in-depth semi-structured interviews with 27 students and 29 clinicians, conducting up to three interviews per participant over a year, resulting in 124 interviews. Participants represented five healthcare professions: medicine, nursing, occupational therapy, physical therapy, and social work. Employing a constructivist grounded theory approach, our data analysis revealed two prominent dimensions: (a) The contextualization of identity formation processes and (b) The identity navigation dimension in which the professional identity and disability identity are explored. This emerging model sheds light on the dynamic processes involved in identity formation, emphasizing the significance of a supportive environment for disabled students and practitioners. Such an environment fosters the negotiation of both professional and disability identities. Moreover, this study recognizes the importance of a re-examination of the concepts of professionalism and professional identity in healthcare professions. In conclusion, this research underscores the importance of understanding and supporting the multifaceted identity formation processes among disabled individuals within healthcare professions.

**Supplementary Information:**

The online version contains supplementary material available at 10.1007/s10459-024-10386-4.

## Introduction

Professional identity (PI) is the set of meanings individuals use to describe how they perceive themselves professionally. PI is a broad concept that includes a body of knowledge, community recognition, group belongingness, a code of ethics, and community norms (Fitzgerald, 2020). Its formation represents the process by which people seek to integrate their various statuses, professional roles, and diverse professional experiences into a coherent image of self. It is also defined as one's professional self-concept based on attributes, beliefs, values, motives, and experiences (Cruess et al., 2015, 2019). Developing a sense of PI is vital to any individual’s educational and professional journey in healthcare professions (Cruess et al., 2019; Jarvis-Selinger et al., 2012).

PI formation in healthcare settings is a dynamic, developmental, and multilayered process. It occurs concurrently at both the individual-psychological level and the collective and interpersonal level and includes individuals’ socialization into their professional roles (Cruess et al., 2019). Within this process, the qualities, ethics, and standards of the healthcare profession are internalized, ultimately impacting individuals’ thoughts, actions, and behaviors that form their role as a healthcare professional (Jarvis-Selinger et al., 2012). In many ways, forming a PI is a process of socialization into a community or a culture, far greater than just ‘professional training’ (Cruess et al., 2019; Matthews et al., 2019). As healthcare students and clinicians repeatedly follow role expectations and enact specific behaviors, the role becomes internalized as part of the self. It is then that the individual moves from ‘performing’ the healthcare professional role to actually ‘being’ a healthcare professional (Jarvis-Selinger et al., 2012).

Healthcare professionals’ PI formation develops in many locations, including classrooms, healthcare facilities, and any areas where students and clinicians have interpersonal interactions. Each setting represents a ‘mini-cultural’ environment where PI formation occurs (Cruess et al., 2015). PI formation is founded on students and clinicians’ individual identities; these significantly influence the type of practitioners they become. However, race, gender, ethnic background, and the larger socio-historical context are often neglected in PI formation research, resulting in the underrepresentation in the literature regarding the development of PI formation processes among equity-denied groups (Fergus et al., 2018; Wyatt et al., 2021). Therefore, in this study we investigate how disabled students and clinicians in healthcare professions—medicine, nursing, occupational therapy, physical therapy, and social work—actively shape their PI and how it affects their disability identity during their educational and professional trajectories.

It is crucial to note that professionalism and professional identity (PI) are constructs that might unintentionally impose regulations or suppress various lived experiences and identities (Bulk et al., 2020; Jain, 2022; Jarus et al., 2022). While often viewed as pillars of the professional world, these constructs could inadvertently oppress and stifle the expression of diverse ways of living and being (Sternszus et al., 2024). For example, a study exploring the experiences of equity-denied groups—such as Black and Indigenous students and clinicians—in forming their PI found it was a challenging negotiation that takes place in a difficult socio-historical context due to experiences of racism and, by extension, a lack of perceived professionalism, within the healthcare system and society at large (Joy-Correll et al., 2022; Wyatt et al., 2020). Therefore, it is imperative to scrutinize how these constructs shape norms and practices within professional settings and how they might inadvertently limit or marginalize individuals who deviate from mainstream expectations.

## Healthcare professional identity and disability identity

Although critical social perspectives are starting to play an important role in the study of PI formation in healthcare professions, little is known about how disabled individuals form their PI in healthcare settings. The healthcare PI formation literature is mainly based on the perspectives of students and professionals *without* disabilities, also known as able-bodied individuals. The disability identity is unique in healthcare-related educational and professional settings. Disabled individuals hold dual roles as both service providers and service users. Consequently, they develop unique perspectives within the healthcare context that are valuable to the field (Battalova et al., 2020; Jarus et al., 2022; Mayer et al., 2022).

However, some challenges have been identified regarding a dual positionality. For example, healthcare professionals may face stigma in the workplace if colleagues have prejudicial beliefs about people with disabilities. In addition, they may spend months navigating unhelpful bureaucracies in an attempt to access accommodations (Gross et al., 2023; Jarus et al., 2022; Mayer et al., 2022; Meeks et al., 2015). While these challenges demonstrate that disability stigma is a social pathology and not a true representation of the disabled individual, society still continues to view dependence and helplessness as synonymous with disability and unprofessionalism (Easterbrook et al., 2015, 2019). Therefore, understanding how disabled individuals navigate integrating a disability identity with PI is imperative in order to identify areas where system change and education is needed.

Disability identity refers to the sense of self in relation to disability and includes feelings of connection to, or solidarity with, the disability community (Forber-Pratt et al., 2017). A coherent disability identity is believed to promote a positive sense of self, enable adaptation to disability in daily life, and expand awareness of and involvement in social activism (Dunn & Burcaw, 2013). Disability identity research is still fairly nascent as research-based literature has only been available from the mid-1990s, not to mention the role of prejudice and bias as negatively impacting the social desirability of research on disabilities. Likewise, there are barriers surrounding the opportunity to study disability and professional identity integration in healthcare fields. For instance, an accurate estimate of disabled individuals in the healthcare fields does not exist. This may be a result of individuals’ hesitance in disclosing their disabilities due to discrimination and the associated stigma of perceived incompetence of disabled individuals (Bulk et al., 2017; Easterbrook et al., 2015, 2019). This estimate becomes even more difficult to establish when considering the presence of invisible disabilities (e.g., chronic pain, mental health disabilities, learning disabilities, etc.). As the disability identity model moves away from a stigma-based understanding towards disability pride, we desperately hope that more disabled individuals will feel comfortable disclosing their status (Forber-Pratt et al., 2017).

There has been growing attention to the potential benefits of healthcare providers living with disabilities in the healthcare arena. For instance, lived experience seems to promote genuine empathy in clinicians living with disabilities while also drawing attention to and challenging social barriers (Battalova et al., 2020). Further, Mogensen and Hu (2019) argued that the perception of disability in the health professions is shifting towards a positive outlook. In their study, 207 participants ranging from 17 to 71 years of age, most of whom knew someone with a disability, were asked about their views on disabled individuals being accepted into medical school. The majority of participants agreed disabled individuals should be encouraged to study medicine and be accepted into medicine school, maintaining the stance that disabled individuals should be able to pursue medicine (or any career) like anyone else (Mogensen & Hu, 2019).

Yet the development of PI and disability identity may sometimes be perceived as two incongruent identities and result in ‘identity dissonance’ (Bulk et al., 2017). In one study, students voiced tensions they grappled with—such as concerns about appearing incompetent or lazy—in relation to their perceived inability to meet the expectations of the ‘good student’ (Bulk et al., 2017). Another study found that trainee identity development can be impeded by a lack of accessibility in healthcare placement settings (Mayer et al., 2022). Moreover, clinical educators’ lack of understanding of how a disability might impact a learner’s work can suggest to disabled learners that their disability makes them a liability, and this, in turn, could impede the development of their PI (Pearlstein & Soyster, 2019; Schwarz & Zetkulic, 2019). Disabled healthcare clinicians and students often struggle to prove their competence because of the stigma attached to their disabilities (Anonymous, 2019; Donohue, 2017; Jarus et al., 2022; Neal-Boylan et al., 2012).

The Social Identity Complexity theory (Roccas & Brewer, 2002) addresses the intricacies of these multiple identities. It does so by describing individuals’ interrelationships among multiple groups identities, such as PI and disability identity, in addition to considering the degree of overlap between groups to which individuals belong. In their study with ten disabled medical students, Stergiopoulos et al. (2018) built on these concepts and found two main findings relating to making sense of identities. One was ‘compartmentalization,’ (Roccas & Brewer, 2002) whereby participants separated their disability and medical student roles. The second, ‘identity intersection’, emphasizes how participants brought together different identities to create an intersectional group membership. This study was one of the first mapping the PI formation of disabled medical students. However, it focused on only one healthcare profession and included a small number of participants.

Since PI is strongly impacted by environmental and social contexts, such as the type of healthcare profession, specific setting (class, clinic, hospital), and stage in education (student, resident, clinician), it is essential to explore the development of healthcare students and professionals’ PI in a diverse sample. The present study included a large sample of disabled students and clinicians from diverse healthcare professions and contexts. Our research questions were: (1) How do disabled students and clinicians in healthcare professions experience PI formation during their educational and practice journey? (b) What are the challenges and benefits or strengths for disabled people in forming their professional identity in healthcare professions alongside their disability identity? These research questions were based upon the belief that understanding the challenges disabled individuals face and strength they bring to the healthcare field can pinpoint where and how disabled individuals require support during the formation of their identities. Further, as the foundations for PI are laid during one’s educational and practice trajectory, support and change may be enacted on an institutional level.

## Method

### Participants

We conducted in-depth semi-structured interviews with 27 students and 29 clinicians (*N* = 56) up to three times over a year for a total of 124 interviews. Participants were disabled students or clinicians in one of five healthcare professions: medicine, nursing, occupational therapy, physical therapy, and social work. Table 1 presents the demographic data of the participants.

**Table 1:** Participants’ characteristics

	Students (*n* = 27) *N* (%)	Practitioners (*n* = 29) *N* (%)	
*Gender*			
Women	16 (59.25%)	19 (65.51%)	
Men	5 (18.5%)	10 (34.48%)	
Other (nonbinary or prefer not to say)	6 (22.22%)		
Type of disability*			
Physical disability (e.g., rheumatoid arthritis, diabetes)	9 (33.33%)	17 (58.62%)	
Sensory disability (e.g., hearing or visual impairment)	5 (18.51%)	7 (24.13%)	
Mental disability (e.g., depression, anxiety, PTSD)	14 (51.85%)	6 (20.68%)	
Cognitive disability (e.g., ADHD, other learning disabilities)	7 (25.92%)	3 (10.34%)	
*When the disability was acquired*			
In childhood or prior to health profession education	21 (77.77%)	16 (55.17%)	
During health profession education	3 (11.11%)	2 (6.89%)	
During health profession practice	1 (3.70%)	8 (27.5%)	
Choose not to report	2 (7.40%)	3 (10.34%)	
* 26 (46.42%) participants reported more than one disability			

This study was conducted in two large Canadian cities (one eastern, one western) and one smaller western city. Participants met the following inclusion criteria: involvement in one of the pre-identified healthcare professions; self-identified as having a disability for at least two years—a portion of which occurred during schooling or practice; for students, experience with both academic and fieldwork; consent to participate. Disability was defined as any physical or mental dysfunction, either officially diagnosed or undiagnosed, including physical disability, mental illness, developmental disabilities, and learning disabilities (Ontario Human Rights Commission, 2016). The work experience of the clinicians ranged from two to 30 years, demonstrating a wide range of experience as a disabled healthcare practitioner.

## Procedure

This study received approval from the universities’ behavioural research ethics boards at the university of British Columbia. Participants were recruited through advertisements on bulletin boards, e-mails sent from their academic departments, and social media posts. Informed consent was acquired from all participants prior to joining the study. A semi-structured interview enabled participants to describe their lived experiences, and included such questions as: ‘How, if at all, does living with a disability relate to your identity?’; ‘How do you feel about the intersection between your disability experience and work/studies experience as a student or clinician?’; ‘Compared to your co-workers or peers who do not have a disability, how, if at all, does having a disability affect your job/studies?’ Participants were interviewed two to three times over one year. All interviews were recorded and transcribed.

## Data analysis

We employed a constructivist grounded theory approach, wherein data collection and analysis co-occurred, and subsequent interviews were partially shaped by emerging ideas as a means to study social processes (Charmaz, 2006, 2016). Team members met to discuss emerging ideas, generate theoretical categories, and explore multiple understandings and interpretations of the data. Rather than relating to frequency, the significance of theoretical categories was determined according to how they contributed to the depth of knowledge. The data were categorized using theoretical sampling, a commonly used method in constructivist grounded theory (Charmaz, 2006). Theoretical sampling involves a process of iterative data collection directed by evolving insight rather than by predetermined categories (Draucker et al., 2007). As theoretical constructs evolve, specific information is sought to refine emerging ideas. Participants were consulted as a means to confirm or reject emerging themes and guide the developing research.

A team of researchers independently analyzed the transcripts using a constant comparative method to generate initial codes, followed by focused coding to specify the to develop and explicate processes. Building on the initial theoretical category understanding, the research team determined the aspects of the interviews most likely to provide the empirical indicators required for theoretical category development. Theoretical saturation was addressed through a representative sample and numbers of interviews collected across three sites. The coding was completed using the NVivo 11 data analysis software. After all the interviews were coded, the transcripts were reviewed to ensure all data referring to theoretical dimensions had been found, and relevant quotations were collected and organized into a table.

## Positionality of authors

The multidisciplinary team included academic researchers: some are healthcare professionals and some have experiential knowledge of disability. As such, we hold diverse perspectives that allowed us to critically explore the data, holding the complexity of thinking and writing about identities and disabilities. Nevertheless, we are firm in our belief that there is no ‘one truth.’ Therefore, we present our findings as a mosaic of perspectives that advance the understanding of how disabled healthcare students and practitioners develop their PI. Following the principles of the second wave of grounded theory, our research team acknowledged that knowledge is constructed and influenced by the social contexts of the researchers themselves (Charmaz, 2016). This goes beyond simply considering one's social position and its effects on the research; it involves a reflexive process where our team negotiated their own perspectives and acknowledged the ever-changing dynamics of power (Hesse-Biber & Piatelli, 2007). Consequently, our team approached the topic with personal significance, understanding that our connections to the research are complex and cannot be neatly categorized as insider or outsider perspectives. While our primary perspective leaned towards constructivism, it is important to note that some of us also brought critical perspectives into play, which were interwoven throughout the analysis and writing processes.

## Findings

In this study it appeared that PI was developed through interactions with others in the education and practice communities, including faculty, professors, supervisors, peers, colleagues, friends, and family. Two main dimensions emerged that build a conceptual understanding of how PI formation occurs within these interactions: 1. The Contextual Dimension, and 2. The Identity Navigation Dimension. The categories and exemplar quotations are presented in Table 2.

**Table 2:** Dimensions and exemplar quotations

Category	Exemplar quotation
1. The Contextual Dimension	
Healthcare settings are competitive, stressful and protocol-driven	“I think I would say, like, if I were to apply to the ER and it is a busy, crazy environment, you have to be on your game.” (Sarah, student) “I feel, because the program is so fast-paced and so competitive to get in, you have to put on this perfect face. And I feel like you can’t let any cracks under your armor show… I still feel like it could be used against you… I always felt I never looked quite good enough there. Everybody was hard on everybody, everybody was really impatient and hard and fast… and I couldn’t keep up.” (Valerie, student) “It’s been really hard because I don’t have any accommodations at all. It’s hard when you’re all in the same boat, right? So all of us in the department are covering for each other so you don’t want to say, ‘well, I can’t cover’. So you try but then I get really tired.” (Rena, clinician) “I feel like I experience disability when people ask me to do things the way everyone else does them. If they asked me to do this, I would do it how I would do it. But when they ask me do this, this way, in this amount of time using this thing, then I feel in certain situations, depending on what those things are, then I may experience disability.” (Jocelyn, student)
Disability Perceived as Weakness	“I don’t want them to treat me differently, singled out for doing poorly.” (Trevor, student) “It [the disability] just gets blown up into this big, oh my gosh, are you—are you gonna be a liability, is this going to go poorly?”—Jocelyn (a student) “It felt like I had to be healthy in order to pursue a healthcare profession.” (Sabrina, student) “Sometimes you have to say—otherwise they’ll think you’re stupid or slow—you have to say, ‘you know, I have a disability and this is what it is’. And it’s hard to say it. It’s so hard… It’s a core belief that I’m not as smart as the others because of my disability.” (Bay, student) “And I struggled because of my anxiety. I struggle, struggle in social situations and so I’ve had a really, really difficult time, um, finding my place and my role on this team in my practicum… I worry that other people are going to think that they shouldn’t be [voice fades away]. That I shouldn’t be in this field because I do struggle so much.” (Ally, student) “If I go into [a] traditional residency system, I will at some point be perceived as weak, and then, sort of pushed aside… the doctor of the day [can say] who is this [health profession] student to think that they can just take breaks!?” (Brianne, student) “I think that’s kind of, like, false strength perhaps. But it is something that kind of like, it’s like, I’m seen as a confident, competent person—despite the fact that I really, like, have ongoing issues with many different things. So I see that kind of being like a toxic reality.” (Luke, clinician)
2. The Identity Navigation Dimension: Patterns of Integration between Professional and Disability Identities	
Compartmentalization	“I think I hold back in that sense, not wanting to use that word [disability] because of the, like I said before, all the issues around it.” (Kiya, student) “I get frustrated… So when people are very focused on it and that’s always what they talk about and what they’re asking about, it drives me nuts cause I’m like, I’m more of a person than just my wheelchair… also see beyond it and see the strengths and capabilities of the person” (Venus, clinician)
Integration—General	“I will continue to be a client along with being a professional, defining my roles at different times with different people.” (Sally, student) “I feel when we talk about disabilities, when we talk about services for disabilities, when we talk about stigmatization, I really empathize and relate to these people—the sort of conflict that they have about disclosing and about how you identify with it and about how you talk to people about it. I get that without having to explain it. I think, for me, that’s how I integrate it.” (Rena, clinician) “I feel like it’s great cause I can draw on my experiences to help the clients.” (Mara, clinician)
Integration—Repression Pattern	“It doesn’t matter if you have things in common with your patients in the future… It could be used against you. If you are gonna be able to provide safe patient care or say, you are not taking care of yourself enough, how do you take care of somebody else? Say, you are on medication, how are the medications going to affect your abilities to work and ability to think and ability to care for other people.” (Valerie, student) “And since I have a diagnosis, I was like, well, I don’t really know if I want to diagnose people. I just, I, like, I know there are, there are definitely benefits, but I don’t, I don’t like that, if you have a disability, you’re kind of the one who had to make an adjustment, it’s not like anybody else [had to].” (Serina, student)
Integration—Extension Pattern	“But also having worked with these difficulties in myself and recognizing them even more and becoming more self-aware allows me to—like, so often I’ll be working with people or children and I’m thinking, ‘I can see where some of these accommodations would help you because they helped me.’ Or, you know, I’m seeing maybe why people are frustrated or things aren’t working. So it gives me a different lens for working with people, which I think is a good thing!” (Sally, student) “Because I’ve had to work so hard to try to understand myself that now I feel like I can use that to relate to people.” (April, student) “In terms of owning my disability and how it comes out in me, that’s part of the reason why I went into [HCP], is that I have an experience of having a disability and I want to help other people.” (Leanna, student) “I feel like people do respect what I do have to say especially when it comes to disability because I feel like I am arguing, always, from a different point than anyone else and I have another level of insight. So, I think I felt like I had some credibility built up to have an opinion on this.” (Jocelyn, student) “But I think it’s [participant’s disability] definitely helped asking questions and being able to make people comfortable and, like, the door’s open. And being able to advocate for people and seeing the side of being a patient as well as being the healthcare provider. Like, being a patient, you see the people that walk into the office and they don’t treat you like a person—you’re just a disease. And now knowing, well, you need to look at the whole person—ask about their stress, ask about their family life.” (Jamie, student) “I think I’m a lot more compassionate for others. Yeah. I have a better understanding of what a disability is.” (Bruce, clinician)

## The contextual dimension

The contextual dimension describes the healthcare context in which the identity formation processes occur and are formed in ways that are bound to social context. The context is especially important when forming PI because professional roles must be legitimized by the group or by the community of practice to support the formation of PI. Participants described a social context where they experienced their professional role was not always legitimized because of their disability. The doubt they experienced from significant others in the healthcare community contributed to hindering participants’ PI formation and sense of belonging to the professional community. This was more prevalent among students because their sense of PI was still in development. Interestingly, despite their diverse contexts, various professions exhibited remarkably similar patterns.

Two prominent categories of the social context contributed to the challenges faced by disabled students and clinicians: (1) healthcare settings are competitive, stressful, and protocol-driven; (2) within these social contexts, disability is, at times, perceived as a weakness and risk to patient safety and professionalism.

### *Healthcare settings are competitive, stressful, and protocol*-*driven*

Healthcare settings are competitive and stressful, and this adds to the challenges experienced by disabled individuals when forming their PI. Healthcare settings are also protocol-driven, making it harder to implement diversity in practice. For example, participants shared teamwork scenarios with expectations that everyone would work based on protocols and contribute equally, even when the system was not adjusted to provide the necessary accommodations, thus inflicting the burden on other team members. Participants shared how onerous it was to address their pain or difficulties without risking being perceived as unprofessional or incompetent in performing their professional roles as expected from all team members. This created a double burden: disabled students and clinicians managed the hardships of their disabilities and were also required to perform as robust, pain-free, and healthy students or professionals as part of their PI formation, even when accommodations were not provided. Participants also described professional expectations as taking a cookie-cutter approach, with little space for working in different and flexible ways to meet the nuanced needs of each individual. Interestingly, no meaningful differences regarding these opinions were found among participants between professions or disability types.

Since the participants believed that specific ways to perform as clinicians were developed based on abled bodies—such as requiring vision capabilities when filling out medical charts with small letters, and requiring cognitive processing and hearing abilities when participating in a team case discussion where a few people speak all at once—it was difficult for them to play these roles in the expected manner; however, disabled individuals can perform these roles with relevant accommodations. This rigidity limited disabled practitioners to only one route to form their PI—practicing the profession in a fixed and unified way, with no room for variations. Disabled healthcare professions students and clinicians disrupted expectations and norms by doing things differently, although still effectively. Yet they said they were perceived as unprofessional and incompetent: “Sometimes you have to say—otherwise they’ll think you’re stupid or slow—you have to say, ‘you know, I have a disability and this is what it is’” (Bay, a student). As a result, participants stated their sense of professionalism, which is integral to forming their PI, was dampened. As PI develops within relational contexts, students and clinicians mold their PI through feedback from key figures in their community of practice. Consequently, even if these perceptions are not fully internalized, they still impose a burden on students and clinicians, who must justify their performance and demonstrate their membership and legitimacy within the profession.

### ‘Disability as liability’: disability is perceived as weakness or a professional risk

The second aspect of the contextual dimension was related to the experience of the participants that disability was often perceived in the healthcare context as a weakness and as undermining professional competence. Consequently, when disabled individuals formed their PI throughout their education, they questioned their competence and belonging as healthcare professionals. Given that a sense of competence and belonging are pivotal to professional identity formation, these challenges significantly impeded participants’ PI development. Alongside their education or work, participants were compelled to defend their methods, challenge stigma and stereotypes, and validate their professionalism. In addition, there was an implicit expectation that healthcare students and professionals should always convey strength, good health, and positive emotions. The participants described this as: ‘professionals should be healthy,’ ‘put[ting] on a perfect face,’ or ‘suck[ing] it up.’

## The identity navigation dimension: patterns of integration between professional and disability identities

**Fig. 1:**
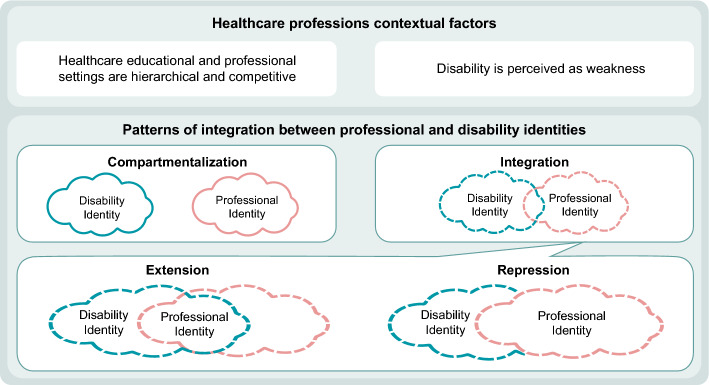
Patterns of integration between disability identity and professional identity

The identity navigation pattern describes the participants’ understanding of the way that their professional and disability identities interact. As demonstrated, PI formation was not a passive process but rather an active one, in which participants constructed and deconstructed their PI within their educational and professional journey. The PI formation took place in both the interpersonal world and in the inner world. Some general patterns of this process emerged as well as several levels of identity navigation between disability and professional identity. In one pattern of integration, participants constructed their sense of who they were as healthcare students or clinicians by merging their disability and professional identities. Another pattern had individuals construct their identity with complete compartmentalization between their PI and their disability identity. See Table 2 for exemplar quotes and Fig. 1 for the illustration of the model.

Two factors appeared to impact the nature of identity navigation. First, the duration of the disability—the longer the experience of the disability, the more it became part of the disabled student’s or clinician’s general personal identity. Living with a disability for longer provided more opportunities to form a disability identity and to incorporate this into an evolving PI. Second, with higher levels of education and more professional experience, the merging of the disability identity and PI was more likely. Students still new to the field, subject to a hierarchical structure in which they are constantly evaluated, and still feeling insecure about themselves as part of the community were more hesitant about integrating their disability identity into their PI. A higher level of experience and longer time living with a disability seemed to interact, such that both led to higher levels of integration. The patterns varied between compartmentalization to integration, while integration could have different presentations as described in Fig. 1.

It is important to note the difference between holding a disability identity and actual disclosure of the identity to others. Some participants identified as disabled but did not outwardly disclose this identity while others both identified and disclosed their disability identities. These types of self-representation encompassed strategies for self-presentation more than an internalized sense of identity. Therefore the data were closely screened to differentiate these two related, yet distinct, constructs.

## ‘Not wanting to use that word—“disability”’: compartmentalization

Compartmentalization was a strategy described by the participants—both students and clinicians—who did not incorporate their PIs with their disability identities, but kept them separate. One explanation for this pattern was the perception that the disability did not integrate well with the healthcare PI. Ally, a student, expressed the compartmentalization pattern: ‘I don’t allow [the disability] to affect my experience with clients…’. One motivating factor in compartmenting the two identities was the perception that integration would be costly, and the disability would color the whole self. As described above, owing to the way in which society perceives disability as a weakness and as undermining individuals’ competence, a compartmentalization pattern may represent the consequences of facing discrimination and negative assumptions, contributing to a sense of separation between the two identities. Some participants did not see any relevant connection between the two, and preferred this identity expression as a personal choice. Some phrases used by participants to describe this identity expression were: ‘It [the disability] is not a big part of me’; ‘My disability is not connected to my profession.’

Externally constructed processes and social expectations lead to internalized stigma. At times, this self-stigma impacted participants, rendering them unable to recognize the strength that disability contributes to serving as a healthcare professional. The lack of legitimacy for disability experiences as professional experiences resulted in fewer opportunities given to disabled individuals to use their disabilities as a professional resource and therefore as an essential part of their PI. This caused frustration, and participants were unable to extend their disability identity to their PI.

## ‘I can draw on my experiences’: integration

Participants shared various levels of integration, connection, and exchange between the two identities, bringing their disabilities forward and drawing on them as part of their professional experience. For example, based on their experiences as patients, disabled professionals understood what would be important from the patient’s perspective and used that information as a healthcare provider. Some participants integrated only small parts of their identities; for instance, they noted how certain aspects of their disability extended their PI but did not feel that the disability colored their entire PI. Others substantially incorporated the two identities and believed their disability identity was a large part of their PI. Some said they decided to pursue a healthcare profession because of their experiences as patients; in other words, their disability became a core component of, and driving force behind, their PI. Participants said their disability experiences added to their knowledge by contributing firsthand experiential knowledge. Others spoke of experiencing greater empathy and being able to understand their clients' experiences from a personal perspective.

## Integration-repression type

Repression was a unique pattern in the data, wherein only a few participants demonstrated this pattern. In this case, participants incorporated their PI and disability identity, but this integration hindered some PI development. Repression was characterized by the internalization of external criticism and discrimination regarding disabilities, hardship, and feelings that some parts of their PI were at risk or must be inhibited in relation to their disability identity. For example, one student described how diagnosing people in general was something she disagreed with as a disabled person. She indicated it was difficult for her to incorporate this into her professional role as a disabled individual, and she had to repress some important aspects of her disability worldviews in order to develop her PI.

## Integration-extension type

Some participants described their PI as enhanced by their disability identity. Integrating the disability experience seemed to contribute to a PI that emphasized a rich, nuanced, complex, and empathic understanding of the client’s experiences. Participants reported their disability identity enriched their PI, added unique and relevant knowledge, and improved patient’s care and relationship building with their clients. Participants conveyed that their firsthand experiences with disabilities were meaningful and unique, informed their PI, and made them better healthcare professionals. These experiences provided them with credibility and additional professional insight that became part of their self perception as professionals.

In addition, participants believed that being disabled healthcare professionals gave them an opportunity to become role models for their clients and their PI was more extensive than that of their peers with no disabilities. One clinician described how, as a student, she was placed in her practicum in the same hospital she was hospitalized in as a child. She said she was empowered by knowing she could now help children with experiences similar to hers. She and other participants described an empowering and positive feeling related to profoundly understanding the experiences of patients and their families, a feeling that enhanced their PI development. By the same token, some thought the reflection processes they engaged in regarding their disability identity were critical assets for them as healthcare professionals.

It is important to note that the pattern of extension sometimes could take an emotional toll in the sense that reflecting on disability experiences while performing a professional role could be triggering and bring up painful emotions. For example, some participants mentioned recalling feelings of helplessness or hopelessness while navigating the healthcare system as a service user. In this sense, at times the participants’ ability to understand their clients’ experiences was an extra challenge.

However, even among participants who expressed extension patterns, disability experiences were not always perceived or credited as valuable professional experiences by others in their communities of practice. The participants believed there were not enough explicit opportunities as they would like to have to express their strengths stemming from disability experiences to the profession, such as invitations to share knowledge or to answer the questions of colleagues who wanted to learn more. At times, perceptions of disability as weakness colored the whole person and the strength that disability brings to a healthcare professional was lost. The lack of legitimacy for disability experiences as a core contributing factor towards professional experiences—such as increased understanding and compassion with clients due to firsthand experiences—resulted in fewer opportunities for disabled individuals to use their disability as a professional resource and as an essential and extended part of their PI.

## Discussion

This study pioneers an exploration of the distinctive challenges and tensions experienced by disabled healthcare professional students and clinicians in shaping their professional identity within academic and workplace settings. Healthcare professions are characterized by their competitive nature as well as explicit and implicit ableism (e.g., Jarus et al., 2022; Stergiopoulos et al., 2018). These factors create barriers and obstacles in developing a sense of belonging for disabled individuals in the healthcare field, creating a challenging climate for disabled individuals seeking to form their PI. The literature generally points to a one size fits all approach: disabled individuals are expected to follow specific procedures that are based on abled healthcare professionals in order to become accepted by the community of practice as a healthcare professional (Jarus et al., 2022; Mayer et al., 2022). Maristany et al. (2023) found that the definition of professionalism among historically marginalized healthcare providers can be an oppressive force. Specifically, tenets of professionalism guide healthcare practitioners but can also limit their personal expressions because the model of the ideal healthcare professional is a white, Western male. In fact, healthcare professionals are often perceived as less professional based on sexual orientation, the presence of religious garments, or how they wear their hair (e.g., dreadlocks) (Maristany et al., 2023).

We discovered a spectrum of patterns between disability and professional identities. Compartmentalization was identified when individuals expressed the need to separate different aspects of their identity. This was not a typical pattern and was identified mostly by students. Another pattern that caused more distress was when participants thought that some parts of the PI had to be repressed in order to stay true to their disability identity. These findings underscore the rigidity inherent in the construct of professionalism and its function as an enforcement and reinforcement mechanism for existing power structures. Present discourse within healthcare education emphasizes the imperative to reconceptualize and reimagine professionalism and its associated norms (Sternszus et al., 2024). The complex process of PI formation encompasses a mixture of tacit and explicit learning, profoundly shaped by social and societal hierarchies. Consequently, there is an urgent need to reconsider the process of PI formation and scrutinize how professionalism is evaluated and determined. Professionalism may act as a mechanism to suppress diverse voices and identities, by prescribing what is deemed “healthy” or “normal.”

It is helpful to compare the patterns found in this study to the four patterns identified in the Social Identity Complexity theory (Roccas & Brewer, 2002), some of which have commonalities with the patterns we found. They described a pattern termed Compartmentalization, wherein multiple identities can be activated and expressed through a process of isolation; a pattern that we too found in our study. This pattern will be present in a person identifying as a student or a clinician and a disabled person but the two social identities are not merged as a means of personal choice and self-expression.

The Social Identity Complexity theory also attends to a pattern called Merger, wherein the social identity is the sum of one’s combined group identifications. While there are some similarities between that pattern and the integration pattern we identified among our participants, it is crucial to note some differences, particularly in the healthcare context. A significant aspect of the healthcare professions involves cultivating a professional identity; it is not solely about merging and embracing two social identities, as described by Roccas and Brewer (2002). It encompasses the development of an entirely new identity alongside the disability identity, and may be still evolving for those who showed this pattern. Hence, we assert that this represents a distinctive status deserving its own terminology. Another pattern identified by the Social Identity Complexity theory is Dominance, which describes adopting one primary group identification to which all other potential group identities are subordinated (Roccas & Brewer, 2002). This shares some commonalities along with some nuanced differences with the Repression pattern found in our data. As described, Repression is characterized by the internalization of external social criticism relating to disabled individuals, especially those in the healthcare professions. In Repression, the integration process impedes the ability of individuals to adopt some aspects of the new healthcare identity that they are being socialized into.

Some of the quotes within the Integration-Extension pattern could potentially be interpreted through the lens of the last pattern identified by the Social Identity Complexity theory—the ‘Engagement status’ (Forber-Pratt & Zape, 2017). However, the integration status is not merely synonymous with engagement status in the healthcare domain. Within healthcare professions, a significant aspect involves cultivating a professional identity. It is not solely about engaging with a specific community; it encompasses the development of an entirely new identity alongside the evolving disability identity. Hence, this represents a distinctive status that we termed as Integration-Extension.

Drawing on the Social Identity Complexity theory, this study emphasizes that the integration of PI and disability identity involves a nuanced negotiation process. Disabled students and clinicians are challenged to determine the extent to which their disability is woven into their professional roles and, if so, the specific dimensions of its inclusion. This is a psychological, social, and ontological process. Participants described layered internal thinking processes, integrating the ways they defined and presented themselves to others and the meanings they made of their behaviors and others’ reactions.

An integral consideration lies in the dynamic interplay between time and the development of PI and disability identity. In their grounded theory study, Forber-Pratt and Zape (2017) discovered four distinct developmental statuses that illuminate the intricate journey individuals with disabilities traverse as their disability identity matures. While our study did not explicitly uncover discernible patterns of change over time, this could be attributed to the specific nature of the questions posed, which may not have elicited reflections of this nature. Nonetheless, cross-sectional comparisons did offer some insight into this aspect, revealing that longer experiences with disability or within the healthcare professions fostered the development of both identities and afforded more opportunities for their integration.

We hypothesize a co-existence of both identities, with each likely exerting influence on the other. This suggests that the presented patterns merely scratch the surface of a reality that is likely far more intricate. In this more nuanced reality, the growth and evolution within each identity likely intersect in a complex interplay, both enriching and challenging each other in ways that defy simplistic categorization. The proposed categorization offers a valuable lens through which to examine the interaction between these two constructs. However, it prompts us to consider a deeper question: What if these are no distinct constructs, but rather facets of a single entity that evolve and develop in tandem? While some hints of this integrated perspective emerge from the data, robust evidence exists for the ways in which participants segmented their experiences of disability and PI. This prompts us to explore further whether these constructs truly exist in isolation or if they are intricately intertwined components of a broader, evolving narrative of identity. Based on our findings, we argue that disabled healthcare students and clinicians have an extra burden of navigating tensions, dilemmas, and inner conflicts when forming their PI in their educational and professional journeys.

The findings of this paper underscore the urgent need for a critical examination of professionalism and professional identity as constructs that may inadvertently serve to police or oppress diverse ways of living and being. However, amidst these challenges, our research offers a glimmer of hope. It suggests that there are opportunities to cultivate more nuanced and multifaceted identities that have the potential to enrich both knowledge and practice within the healthcare field. Importantly, our findings highlight the crucial role of support in this endeavor. It can be argued that disability often arises from an environment that fails to accommodate the needs of individuals. In essence, disability is not solely a result of individual limitations, but rather a reflection of institutional barriers that hinder the efforts of disabled individuals. Therefore, our study emphasizes the significance of fostering supportive environments for disabled individuals within healthcare professions to nurture their professional identity. A nurturing learning environment, we argue, will facilitate the negotiation of these two identities, acknowledging the profound opportunities for the healthcare field to benefit from the unique perspectives of disabled clinicians. By embracing diverse identities, healthcare institutions can unlock untapped potential and foster a more inclusive and equitable future for both healthcare providers and service users. Other studies have demonstrated disabled students and clinicians can provide new perspectives, share experiential firsthand knowledge, and bring deep sensitivity and empathy to the encounter with patients (Battalova et al., 2020). These advantages can serve to challenge the current notions of professionalism in the healthcare professions. Our findings provide a foundation for further exploration. Future research could delve into the negotiation of additional intersectional identities, potentially examining multiple layers, such as the experiences of non-white disabled practitioners in comparison to their white counterparts.

## Limitations and implications

This study has important implications but is not without limitations. As it was conducted in two Canadian provinces across three campuses, we do not know about the lived experiences of other disabled healthcare students and professionals at other sites across the country. Further, participants solely consisted of individuals who were willing to disclose their disability identity to the research team. As mentioned above, there is no accurate estimate of how many disabled individuals exist in the healthcare professions due to the stigma surrounding disclosure. Thus, individuals who were not comfortable disclosing their disability identity were not represented in the study. At present, we are unsure what “professional” looks like among healthcare students and professionals as understood in the context of disability and not in comparison to non-disabled students and professionals. We encourage future studies to research this construct and create a new understanding of what professionalism, in the lens of diversity and social justice in the healthcare field, means.

Lastly, while we acknowledge that our work has contributed to a novel understanding of PI development among disabled individuals, we recognize that the extent to which it qualifies as a fully-fledged theory may be debatable. We have introduced innovative perspectives on the role of context and the dynamics of integration in shaping PI. Our study has advanced theoretical discourse in this area and lays the groundwork for future theoretical development. The salient patterns we identified may help educators, supervisors, advisors, disabled students, and clinicians find ways to support disabled students’ PI. The results do not indicate a preferred or predictable pattern, nor do they lead to the creation of a checklist of prescriptive recommendations. Rather, they indicate the nuanced strategies students and clinicians use to negotiate their disability identity and their healthcare PI. Our findings stress the importance of social context in supporting the process of disabled students' and clinicians' PI formation.

The findings of this study underscore the pressing need to examine prevailing concepts of professionalism and professional identity that perpetuate epistemic injustices (Battalova et al., 2020). They advocate for the emergence of more equitable paradigms, redefining what constitutes professionalism and embracing diverse approaches to healthcare practice. This study prompts a critical revaluation of established norms, advocating for justice, inclusivity, and equity in defining the roles and behaviors of healthcare professionals. Since negotiating the two evolving identities could add to the challenges that disabled individuals experience in their training and work, a supportive environment may help them find the best way to safely explore their disability identity alongside their PI. Based on prior knowledge and insights gleaned from the data analysis we created a list of recommendations (see Table 3). A supportive environment can reduce disabled individuals’ experiences of discrimination for not fitting the capability imperative: “a cultural force, or logic, that sets expectations around performance and behavior for those within and who aspire to the medical profession” (Jain, 2022, p. 1).

**Table 3:** Ways to support PI formation of disabled students in healthcare education

1. Acknowledge the ways that PI and professionalism are perceived do not allow much space for diversity. Support disabled individuals and allow for diverse methods of providing care.
2. Professors, preceptors, fieldwork educators, and supervisors should approach their work collaboratively while practicing humility, curiosity, respect, flexibility, and critical thinking when teaching healthcare professions.
3. Create spaces for disabled students and clinicians to discuss their PI formation and consider both professional and disability identities. Allow for critical discussions on healthcare constructs, such as professionalism, and how they replicate power structures that may oppress historically equity-denied groups.
4. Create explicit educational opportunities for disabled individuals to share their experiences so student peers, colleagues and supervisors can learn from them and use this knowledge to improve patient care. Consider experiential knowledge as significant and important knowledge.
*5.* Raise awareness among healthcare professionals, educators, and licensing bodies on disabilities, and educate them about the unique and important contributions of people with disabilities to healthcare education and practice, especially to group competencies.
6. Encourage critical discussions on the concept of professionalism and professional identity and educate healthcare educators and supervisors to allow more complex multidimensional professional identities that acknowledge the contribution of disability experiences and the diverse ways to provide healthcare services.

When considering “safety discussions” it is important to acknowledge that they may hold a nuanced yet profound act of oppression, one that necessitates confronting through a pedagogy of disruption. Disruption of a discourse may at times not be repressive but rather humanizing, offering a transformative alternative (Leonardo et al., 2010). Hence, it is imperative to recognize the dialectic relations between “safety” and “disruption”. The creation of spaces where people with disabilities feel safe may necessitate disruptive discourse especially on concepts considered the bedrock of healthcare professions (Leonardo & Porter, 2010). The adoption of a new, more inclusive stance can overcome the sense that disclosure of a disability within clinical practice is a weakness, flaw, or liability (Jarus et al., 2022; Stergiopoulos et al., 2018). It is essential to create a culture in healthcare professional education programs, workplaces, and licensing boards that not only respects diversity and inclusion, but also actively supports it. The healthcare field should actively cultivate a culture where disability is not downplayed but recognized and valued as a distinctive and enriching life experience. Such acknowledgment can significantly contribute to fostering a diverse and vibrant student and healthcare provider community.

## Supplementary Information

Below is the link to the electronic supplementary material.

Supplementary file1 (DOCX 32 KB)

## Data Availability

The data supporting this study’s findings are available from the corresponding author, but restrictions apply to the availability of these data.
