# Supplementary material for: Belonging in dual roles: exploring professional identity formation among disabled healthcare students and clinicians
Source: Adv Health Sci Educ Theory Pract. 2024 Nov 7;30(4):1101–21. doi: 10.1007/s10459-024-10386-4 (PMC12390862; doi:10.1007/s10459-024-10386-4)
Supplement: Supplementary file 1 — Supplementary file1 (DOCX 32 KB) [file 10459_2024_10386_MOESM1_ESM.docx]

**Appendix 1: Interview Guide**

**Interview I**

Hello. Thank you for agreeing to participate in our study. My name is _______ and I’m a researcher at the University of British Columbia. We are conducting interviews to learn more about the barriers and facilitators to access in education and practice for students and clinicians with disabilities in the health and human service disciplines. Our study involves conducting three interviews with students and clinicians over the next year as well as asking interviewees to refer stakeholders who they think might enrich our understanding and would like to be interviewed.

This interview will last approximately 60 minutes. If you need a break during the interview, please let me know. I would also like to audio record our conversation so that we can interpret the information at a later point. Everything we discuss is confidential and your identity will remain anonymous. When we use information from your interview, we will never use any identifying information, such as your real name or student number. If I ask a question that you don’t feel comfortable answering, we can skip it. You can also end the interview at any time. However, we would greatly appreciate your assistance in helping us better understand the experiences of students and clinicians with disabilities.

Do you have any questions before we begin? (***Begin recording after this question***)

Let’s start by discussing your background.

**I: Demographics/Background Information**

1. Please tell me about where you grew up. (PROBE: Born in Canada? How long have you lived here? etc.)
2. What is your current relationship status? (PROBE: If in a relationship or married, for how long? Live together? Children?)
3. How would you describe your racial/ethnic background?
4. Tell me about your educational background.
5. Tell me about your current occupation. (PROBE: Full time or part-time? Job description)
   1. What motivated you to pursue this profession?
   2. What brought you to this workplace?
6. Do you volunteer?

**IF YES:** What type of volunteering do you do? How many hours do you volunteer each week?

**IF NO:** Ask next question

**II: Disability**

1. Could you please tell me about your disability? (PROBE: length of time with it, visible/invisible, multiple)
2. Could you tell me about the process of receiving your diagnosis? (PROBE: Did you even get one? Was it straightforward, did diagnosis change or develop, etc.)

**If no diagnosis, how do you define your disability? Why didn’t you receive a diagnosis? How does it affect your experiences as a student without a diagnosis?*

1. How, if at all, does living with a disability relate to your identity? (PROBE: Identity change? Feel differently about yourself)

**III: Transition for clinicians**

1. Tell me about your experience as a student.
2. Tell me about your experience as you transitioned from school to working as a health professional. (PROBE: challenges, barriers, facilitators)
   1. What challenges did you experience during this transition?
   2. What facilitated this transition?
3. What was your experience with job interviews like? (PROBE: Did the interviewers know about your disability?)
4. How did your education prepare you for your current job?
   1. Please tell me about any ways in which your education did not adequately prepare you.
5. What advice would you give current students with disabilities as they prepare to transition from university to work?

**IV: Experience as a Student/Clinician in General**

1. What aspects of your studies/job have you enjoyed the most so far? (PROBE: Why?)

1. What aspects of your studies/job have you least enjoyed? (PROBE: Why?)

**V: Experiences as a Student/Clinician with a Disability**

1. How, if at all, has your disability influenced your **experience at your studies/job**? (*PROBE: for* ***specific*** *challenges/barriers/facilitators and then for each one ask the following questions*)

**IF A CHALLENGE:**

- 1. What, if any, aspects of this challenge have you been able to address?
     1. What, if anything, helped you address this challenge? (PROBE: Strategies used? Did someone offer help? Did you ask for help? Why didn’t you ask for help? Who helped? etc.)
  2. What, if any, aspects of this challenge have you been unable to address?
     1. What, if any, barriers have you faced in addressing this challenge?
     2. Who or what could help you with these challenges?

**IF A FACILITATOR**:

- 1. How has this helped you with your training?

1. What, if any, accommodations do you require at your studies/work? (PROBE: Does he/she receive these accommodations when needed?)
   1. Are there accommodations you need but aren’t receiving?
2. How much, if any, control do you feel you have over the accommodations you receive?
   1. What is your involvement in the decision making process? (PROBE: Ask for specific examples and experiences)
   2. What is your involvement in receiving accommodations? (PROBE: Ensuring you receive them?)
   3. What do you believe clinicians with disabilities need to do in order to receive accommodations?
   4. What do you believe educational programs/organizations/workplaces need to do to support the participation of students/clinicians with disabilities?
3. What has your experience with peers/coworkers been like?
   1. How, if at all, has your disability affected your experiences with your peers/coworkers?
4. What has your experience with clients been like?
   1. How, if at all, has your disability affected your experiences with clients?
      1. Do you feel there is a blurring between client and clinician because of your disability?
5. Please tell me about expectations you had about your education/job before you entered. (e.g., about coworkers, about the work itself, about supervisors, etc.).
   1. How have these expectations been met?
   2. How have these expectations not been met?
6. Some professionals report competing demands between supporting practitioners with disabilities and protecting the professional practice. What do you think about this?

**VI: Disclosure of Disability**

1. What has your experience with disclosure been like?
   1. What, if any, are the benefits of disclosing your disability to a supervisor/co-worker/client? (ASK ABOUT EACH GROUP SEPARATELY IF NEEDED)
   2. What factors, if any, limit your disclosure to those people? (Detriments)
   3. Tell me about a time when you told a professor/supervisor/co-worker/client?
      1. How, if at all, did their reaction influence your thoughts regarding telling others about your disability?
      2. Have you ever felt forced to disclose? Explain.
   4. Of the professors/supervisors/coworkers/clients who know about your disability, have any treated you differently? (PROBE: How so?)

**VII: Comparison to peers/co-workers**

1. Compared to your peers/co-workers who do not have a disability, how, if at all, does having a disability affect your job?

**VIII: Plans and goals**

1. What are your goals related to your professional practice?

**IX: Closing**

1. What motivated you to participate in this study?
2. Is there anything you’d like to add regarding your experience as a student/clinician with a disability?

Thank you so much for your time and input.

**Interview Guide II**

Hello. Thank you for agreeing to participate in the second interview. As I mentioned last time, my name is _______ and we are conducting interviews to learn more about the barriers and facilitators to access in education and practice for students and clinicians with disabilities in the health and human service disciplines. Our study involves conducting three interviews with students and clinicians over the next year as well as asking interviewees to refer stakeholders who they think might enrich our understanding and would like to be interviewed.

This interview will last approximately 60-90 minutes. If you need a break during the interview, please let me know. I would also like to audio record our conversation so that we can interpret the information at a later point. Everything we discuss is confidential and your identity will remain anonymous. When we use information from your interview, we will never use any identifying information, such as your real name or student number. If I ask a question that you don’t feel comfortable answering, we can skip it. You can also end the interview at any time. However, we would greatly appreciate your assistance in helping us better understand the experiences of students and clinicians with disabilities.

Do you have any questions before we begin? (***Begin recording after this question***)

**I: Summary of Last interview**

1. When we last met, we discussed several topics, including (give summary of the topics). Do you have any further thoughts or reflections regarding what we discussed last time?

**II: Changes since Last Interview**

1. Have there been any changes in your living situation since the last time we met (e.g., place of residence, relationships status, etc)? If so, have the changes affected your work in any way?
2. Have there been any changes related to your education/work? If so, how did they affect you? (Try to situate this question within the context of the previous interview. E.g., Last time we spoke about….).
3. Last time we met you provided information about your disability. Could you please tell me about any changes related to your disability since we last spoke? (PROBE: Exacerbation, deterioration, new symptoms, improvements, new diagnoses)
4. Were there any changes to the accommodations you receive at work since we last spoke?

**III: Experiences as a Student/Clinician with a Disability**

1. Do you feel that your accomplishments and abilities/strengths have been acknowledged at your program/workplace? Tell me about your own experience with this.
2. Tell me about how, if at all, you’ve ever felt that others have minimized your experiences as a student/clinician with a disability. Tell us about your experiences with this. (e.g., misunderstood the impact of the disability on your life).
3. Tell me about how, if at all, you’ve ever felt that you have to prove your legitimacy as a student/clinician because of your disability. (PROBE: What does this process entail?)
4. What would you suggest for improving the experiences of students/clinicians with disabilities at their workplaces?

**IV: Disclosure of Disability**

1. Since we last spoke, have there been any changes in the disclosure of your disability? (PROBE: Since then have you disclosed to coworkers/managers? Why or why not?)
2. Please tell me about the process of deciding whether or not to disclose your disability? (PROBE: characteristics of the relationship, length of time known the person)
3. How do you explain your disability to others?
   1. What do you explain about your disability to others?
   2. What approaches do you use when you disclose your disability? Please give us an example.
   3. What expectations do you have regarding how others will react to your disclosure?
4. Some students/clinicians mentioned having or not having control over disclosure – can you tell us about your experience with that? How much control do you feel you have over disclosure? (PROBE: do you ever feel forced to disclose?)

**V: Support and Social Support Map**

1. Please take a look at this social support map that we created from your responses to the survey. (*Use this as an opportunity to probe into the relationships with individuals on the map. Ask plenty of follow ups and see where the conversation takes you*).
   1. Does this look right to you? Does anything look off; are there any surprises?
   2. Looking at the support map, what strikes you the most?

Is there anything you would like to change? (Probe: physical distance of the people on the map relative to you, relative to each other; any links between the people that are not marked…)

1. Looking at your social support map, I see that you named ____________ and ____________ as your two most supportive people for carrying out your role as a clinician with a disability.
   - 1. Why do you rely on these people the most?
     2. What qualities do they have that make them supportive? Can you give an example of how they support you?
2. Looking at your social support map, I see that you named _____________ and ______________ as the most hindering people for matters related to carrying out your role as a clinician with a disability. *(NOTE: potentially emotionally triggering)*
   - 1. What makes these people hindering?
     2. Can you give me an example of a time when you were hindered?
3. What are the most important qualities of a supportive person in general?
4. What are the qualities of hindering people, in general? *(NOTE: potentially emotionally triggering)*
5. Do you feel that you are well-supported by your social network? Why or why not?
6. How do you think you could be better supported by your social network?
7. In considering your overall social support network, do you have any thoughts about the way power dynamics in these relationships may shape your perception of being supported or not supported? Tell me more about that.
8. Educational programs/workplaces often discuss the importance of including people with disabilities through offering them support. What, if anything, do you think needs to be changed for your workplace to better support you?

**VI: Advocacy**

1. Some students/clinicians with disabilities feel that they have to invest energy in advocating for their accommodations within their workplaces because structures within the workplace limit their control. How do you feel about this statement?
2. Have you ever had to advocate for yourself at your program/current job? If so, please tell me about it. (PROBE: Tell me about the process, did you speak up against someone who has more power than you? Try to get at how the person advocated… give examples, do you ever feel you have to advocate even if you don’t want to?).
   1. If you have not had to advocate for yourself, please tell me why you don’t think you have had to advocate at your workplace? (E.g., lots of support and accommodations, doesn’t disclose, etc.)
3. How, if at all, is advocacy a benefit to you as a student/clinician? (E.g., helps get accommodations)
4. How, if at all, could advocacy be a detriment to you as a student/clinician? (E.g., rocking the boat)

**VII: Stigma**

1. People with disabilities often face social stigma towards disability. Stigma is defined as any negative attitude or belief that leads people to avoid, discredit, fear, marginalize, or reject those who are seen as being different. What is your experience with stigma? (peers, co-workers, clients, staff, supervisors)
2. What is the nature of the stigmatization that you experienced?
3. What characteristics or traits do people assume you have because you have a disability?
4. How, if at all, does stigmatization impact you?
5. Self-stigma occurs when people internalize negative stereotypes to themselves. What is your experience with self-stigma? *(potentially triggering)*
   1. How do you deal with situations where you feel that you are self-stigmatizing?

25. What do you think students/clinicians with disabilities can do to challenge stigma in their program/workplaces?

a. What can professors, peers, colleagues, supervisors, and others do to challenge stigma in the program/workplace?

**Interview Guide III**

Hello. Thank you for agreeing to participate in the third interview. As I mentioned last time, my name is _______ and we are conducting interviews to learn more about the barriers and facilitators to access in education and practice for students and clinicians with disabilities in the health and human service disciplines. Our study involves conducting three interviews with students and clinicians over the next year as well as asking interviewees to refer stakeholders who they think might enrich our understanding and would like to be interviewed.

This interview will last approximately 60-90 minutes. If you need a break during the interview, please let me know. I would also like to audio record our conversation so that we can interpret the information at a later point. Everything we discuss is confidential and your identity will remain anonymous. When we use information from your interview, we will never use any identifying information, such as your real name or student number. If I ask a question that you don’t feel comfortable answering, we can skip it. You can also end the interview at any time. However, we would greatly appreciate your assistance in helping us better understand the experiences of students and clinicians with disabilities.

Do you have any questions before we begin? (***Begin recording after this question***)

**I: Summary of Last interview**

1. When we last met, we discussed several topics, including (give summary of the topics). Do you have any further thoughts or reflections regarding what we discussed last time? Is there anything in particular you would like us to talk about together today?

**II: Changes since Last Interview**

1. Have there been any changes in your living situation since the last time we met (e.g., place of residence, relationships status, etc)? If so, have the changes affected your work in any way?
2. Have there been any changes related to your studies/work? Tell me about these. What have they meant to you? (Try to situate this question within the context of the previous interview. E.g., Last time we spoke about….).
3. Last time we met you provided information about your disability. Could you please tell me about any changes related to your disability since we last spoke? (PROBE: Exacerbation, deterioration, new symptoms, improvements, new diagnoses)
4. Were there any changes to the accommodations you receive at work since we last spoke?

**III: Experiences as a Student/Clinician with a Disability**

1. What has been the greatest barrier that you’ve overcome in your studies/work experience at _______?
   1. Have you been able to overcome this barrier? Can you tell me more about your experience with the barrier?
2. What has been the greatest facilitator to your studies/work that you experienced _______? Tell me more about this.
3. Most Health and Human Service programs aim to train generalists, rather than specialists. As a clinician with a disability, what do you think about this statement? (PROBE: how did this orientation to training generalists impact your career choices?)
4. Please tell me about balancing work and your personal life with the needs of your disability.

**IV: Role Models and Mentorship *(NOTE: If didn’t ask participants why they chose their careers in previous interviews, be sure to do it now)***

1. Please tell me about any role models you have/had, if any, regarding being a student/clinician with a disability.
   1. What role models exist for practitioners with disabilities (e.g., in media or in daily life)?
   2. What characteristics would make someone a good role model for practitioners with disabilities?
2. Tell me about your experience either being mentored or mentoring someone else with a disability.

**V: Responsibilities**

1. What do you need to do as a student/clinician with a disability to address your needs?
   1. What are your program’s/workplace’s responsibilities regarding addressing your needs?
   2. What are the responsibilities of the University/professional associations? The colleges/registrars?
2. Is there any one competency that is so essential to your profession that if it cannot be accommodated you cannot practice without? Something every practitioner must be able to do? For example, is there a skill everyone must have?
   1. What is this skill?
   2. Is there anything that makes demonstrating this specific skill most difficult for you?
   3. Can you think of an example where someone with a disability would not be able to meet essential competencies?

**VII: Disability Identity and Intersectionality**

How would you define disability? Please give some examples.

1. People with disabilities often face social stigma towards disability. Stigma is defined as any negative attitude or belief that leads people to avoid, discredit, fear, marginalize, or reject those who are seen as being different. What is your experience with stigma? (peers, co-workers, clients, staff, supervisors)
2. What is the nature of the stigmatization that you experienced?
3. What characteristics or traits do people assume you have because you have a disability?
4. How, if at all, does stigmatization impact you?
5. People often experience stigma and discrimination because of disability. But we know this can be compounded if they have other characteristics related to race, sexual orientation, income, gender, etc. Has this been an issue for you?
   1. If so, how are these related to your experiences as a person with a disability?

**VIII: Closing**

1. Since our first interview how, if at all, has the way that you conceptualize or perceive your disability changed (PROBE: as related to identity, peers, work/school)?
2. Reflecting back on some of the barriers that you’ve mentioned in previous interviews, how would you approach them knowing what you know now?
3. What have you learnt from participating in this study?
   1. Is there anything you would like to add that we didn’t ask you?
4. Knowing what you know now about being a student/clinician with a disability in a healthcare profession, what advice would you have given yourself when you started your career at ___?
   1. What advice would you give a new employee starting your job?

Thank you so much for your time and insights.
